# Supplementary material for: Mitochondrial DNA copy number variation across human cancers
Source: eLife. 2016 Feb 22;5:e10769. doi: 10.7554/eLife.10769 (PMC4775221; doi:10.7554/eLife.10769)
Supplement: Figure 3—source data 1. — Third column indicates whether tumor mtDNA content or log ratio of tumor to normal mtDNA content was used. p-values are uncorrected for multiple hypothesis testing. DOI: http://dx.doi.org/10.7554/eLife.10769.007 [file elife-10769-fig3-data1.zip › Figure_3_Source_data_1.pdf]

Name	Cancer	DataType	StromalR	StromalP	ImmuneR	ImmuneP	N
BLCALog2FC	BLCA	Log2FC	-0.04	8.76E-01	0.06	8.06E-01	21
BRCALog2FC	BRCA	Log2FC	-0.13	4.30E-01	-0.18	2.79E-01	38
COADLog2FC	COAD	Log2FC	-1	NAN	-1	NAN	2
HNSCLog2FC	HNSC	Log2FC	-0.04	7.39E-01	-0.08	5.31E-01	68
KIRCLog2FC	KIRC	Log2FC	-0.03	6.43E-01	-0.13	7.36E-02	185
LUADLog2FC	LUAD	Log2FC	-0.32	1.41E-02	-0.34	8.70E-03	60
UCECLog2FC	UCEC	Log2FC	-0.43	9.44E-02	-0.42	1.07E-01	16
BLCATumorMTDNA	BLCA	TumorMTDNA	-0.26	1.34E-02	-0.26	1.42E-02	91
BRCATumorMTDNA	BRCA	TumorMTDNA	-0.2	3.97E-03	-0.15	3.11E-02	205
COADTumorMTDNA	COAD	TumorMTDNA	-0.42	2.29E-01	-0.82	3.81E-03	10
GBMTumorMTDNA	GBM	TumorMTDNA	0.03	7.57E-01	0.04	7.07E-01	117
HNSCTumorMTDNA	HNSC	TumorMTDNA	-0.15	1.68E-02	-0.25	6.53E-05	259
KIRCTumorMTDNA	KIRC	TumorMTDNA	-0.05	4.95E-01	-0.13	5.63E-02	216
LUADTumorMTDNA	LUAD	TumorMTDNA	-0.22	1.26E-03	-0.15	3.19E-02	203
OVTumorMTDNA	OV	TumorMTDNA	0.6	4.00E-01	-0.4	6.00E-01	4
UCECTumorMTDNA	UCEC	TumorMTDNA	-0.15	5.05E-02	-0.18	1.51E-02	179

Table 1: Figure 3 - source data 1
