# Supplementary material for: Mitochondrial DNA copy number variation across human cancers
Source: eLife. 2016 Feb 22;5:e10769. doi: 10.7554/eLife.10769 (PMC4775221; doi:10.7554/eLife.10769)
Supplement: Figure 5—source data 1. — Only cases with at least 20 samples were evaluated. Approximately 35% of studies show statistically significant positive correlation. Log10 range indicates the number of orders of magnitude separating the maximal and minimal value of mtDNA copy number in that cohort. DOI: http://dx.doi.org/10.7554/eLife.10769.015 [file elife-10769-fig5-data1.zip › Figure_5_Source_data_1.pdf]

Name	Spearman ρ	P Value	BH-Adjusted P value	Number of Samples	Log10 Range
ACC Tumor	0.107	3.59e-01	5.59e-01	75	0.747
BLCA Tumor	0.034	5.98e-01	7.10e-01	236	1.067
BRCA Tumor	0.224	3.77e-09	9.81e-08	681	1.462
BRCA Normal	-0.245	3.98e-02	1.02e-01	71	0.708
CESC Tumor	0.196	6.89e-03	2.20e-02	190	1.420
ESCA Tumor	0.408	8.49e-05	4.42e-04	89	0.722
GBM Tumor	-0.059	4.93e-01	6.41e-01	138	0.675
HNSC Tumor	0.165	4.72e-04	2.04e-03	444	1.269
HNSC Normal	-0.152	3.54e-01	5.59e-01	39	0.433
KIRC Tumor	0.113	4.31e-02	1.02e-01	319	1.336
KIRC Normal	0.143	2.54e-01	4.72e-01	65	0.382
KIRP Tumor	0.275	9.59e-04	3.56e-03	142	0.870
KIRP Normal	0.075	6.95e-01	7.52e-01	30	0.289
KICH Tumor	0.193	1.20e-01	2.40e-01	66	0.709
KICH Normal	0.188	3.65e-01	5.59e-01	25	0.453
LIHC Tumor	0.114	1.16e-01	2.40e-01	191	0.872
LIHC Normal	-0.075	6.13e-01	7.10e-01	48	0.466
LGG Tumor	0.126	7.61e-03	2.20e-02	448	0.606
LUAD Tumor	0.197	4.03e-05	2.62e-04	429	1.414
LUAD Normal	-0.109	4.39e-01	6.00e-01	52	0.508
PRAD Tumor	0.032	6.28e-01	7.10e-01	233	0.736
PRAD Normal	-0.004	9.82e-01	9.82e-01	37	0.443
SKCM Tumor	0.091	4.16e-01	6.00e-01	81	1.419
THCA Tumor	0.198	3.42e-05	2.62e-04	434	1.067
THCA Normal	-0.041	7.55e-01	7.86e-01	59	0.498
UCEC Tumor	0.300	4.39e-06	5.70e-05	228	1.427

Table 2: Figure 5 - source data 1
