# Supplementary material for: Mitochondrial DNA copy number variation across human cancers
Source: eLife. 2016 Feb 22;5:e10769. doi: 10.7554/eLife.10769 (PMC4775221; doi:10.7554/eLife.10769)
Supplement: Figure 7—source data 1. — The H-score for KIRC was significantly less than matched normal renal parenchyma in 3/3 samples. The H-score for KIRP was marginally lower than the H-score than matched normal renal parenchyma in 2/3 samples.The H-score for BLCA was higher than the H-score for matched normal urothelial tissue in 2/3 samples. DOI: http://dx.doi.org/10.7554/eLife.10769.020 [file elife-10769-fig7-data1.zip › Figure_7_Source_data_1.pdf]

Tumor Type	Sample Number	Tumor Score	Normal Score
KIRC	1	53.5	225.7
KIRC	2	38.4	199.3
KIRC	3	137.8	216.1
KIRP	1	242.7	215.3
KIRP	2	249.0	256.0
KIRP	3	207.0	253.8
BLCA	TCGA-DK-A1A3	69.8	16.5
BLCA	TCGA-DK-A3IN	141.1	223.3
BLCA	TCGA-DK-A3IQ	152.8	116.7

Table 3: Figure 7 - source data 1
